# Characterization of Modified DNA-Based Polymer Alignment Layers for Photonic Applications

**DOI:** 10.3390/ma18122760

**Published:** 2025-06-12

**Authors:** Rafał Węgłowski, Mateusz Mrukiewicz, Dorota Węgłowska, Malwina Liszewska, Bartosz Bartosewicz, Adrian Chlanda, Anna Spadło

**Affiliations:** 1 Faculty of Advanced Technologies and Chemistry, Military University of Technology, Gen. S. Kaliskiego 2, 00-908 Warsaw, Poland; mateusz.mrukiewicz@wat.edu.pl (M.M.); dorota.weglowska@wat.edu.pl (D.W.); malwina.liszewska@wat.edu.pl (M.L.); bartosz.bartosewicz@wat.edu.pl (B.B.); anna.spadlo@wat.edu.pl (A.S.); 2 Łukasiewicz Research Network—Institute of Microelectronics and Photonics, Wólczyńska 133, 01-919 Warsaw, Poland; adrian.chlanda@imif.lukasiewicz.gov.pl

**Keywords:** liquid crystal, biointerface, biopolymer, anchoring energy, DNA

## Abstract

We present the creation of an alignment layer for liquid crystal molecules based on DNA from fish waste and a selected cationic surfactant. The implemented biodegradable DNA-based surface offers excellent optical and physical properties, cost-effectiveness, and environmental benefits compared to conventional polymers. Our findings demonstrate that the biopolymer DNA-DODA effectively induces homeotropic alignment of nematic liquid crystals, which was confirmed by topography visualization using atomic force microscopy, macroscopy, and polarizing optical microscopy observations. Anchoring energy and response time studies in the well-known electro-optical effect show that DNA-DODA exhibits molecular interaction strengths comparable to those of commercial polyimide. The successful implementation of DNA-DODA as an alignment layer highlights its promise for next-generation technologies, including flexible, sustainable, and biocompatible optical devices.

## 1. Introduction

Aquatic food is a crucial source of protein for human consumption. Depending on the species and processing methods, about 20 to 80% of the total fish body can be considered fish waste [1]. The Food and Agriculture Organization (FAO) reported in 2022 that total fisheries and aquaculture production will reach 15% by 2030 [2]. Primarily, farmed Atlantic salmon has been one of the significant contributors to growth in the global trade of fisheries and aquaculture products in recent decades. Additionally, the FAO is committed to the Blue Transformation strategy as a vision for sustainably transforming aquatic food systems, a recognised solution for food and nutrition security and the environment involving preserving aquatic ecosystem health, reducing pollution, protecting biodiversity, and promoting social equality [2]. Fish waste is out of the reutilisation process but is instead landfilled, incinerated, and discharged into the sea. Those disposal methods can create secondary pollution, an environmental burden and a severe economic crisis. There is a real need to find environmentally acceptable ways of reusing fish waste [3].

One of the possibilities for utilising fish waste by-products is related to synthesizing biopolymers, which can be functionalized for specific photonic applications [4]. Advances in producing biopolymers through chemical, microbial, or enzymatic processes increase their acceptability for commercial usability. The utilisation of fish waste as a bio-based polymer has remarkable potential due to its non-toxic, biocompatible, and biodegradable nature. It has gained a wide audience as a sustainable alternative to petroleum-based polymers. The global production of biopolymers from various organic wastes is expected to rise to 7.5 million tonnes in 2026, and by 2050, global production is predicted to triple [5,6,7].

Commercial polymers are high-functional materials used in specific technological areas, particularly microelectronics applications, semiconductors, and displays. The manufacturing technology of many optical devices is based on a thin polymer alignment layer for organic molecules. The alignment layer is considered a thin film, and the technology is crucial for the global quantity of produced electronic displays. Further, the increasing demand for commercial displays across a wide range of sectors is driving the growth of the retail display market. Infrastructure development, industrialisation, and urbanisation are key factors governing the growth of the global commercial display market, which is expected to be around USD 297 billion by 2030 and expand by 7.35% from 2022 [8]. Polyimides (PIs), comprising highly polarized moieties and electron-withdrawing groups, show a high refractive index and good transparency, with a high potential for such optoelectronic devices, but are not produced by environmentally friendly methods [9]. Therefore, it is essential to obtain polyimides based on non-toxic, bio-renewable raw materials and less toxic conditions for synthesizing this type of polymer.

Excellent candidates for substituting PI layers are biopolymers, such as deoxyribonucleic acid (DNA) [10,11]. This specific biopolymer is under intensive research for many applications, especially outside the biological field of interest [12,13,14,15]. Some of the research is concentrated on the form of DNA–cationic surfactant complexes [16,17,18]. The structure consists of a single or double strand of DNA and different surfactants attaching, through electrostatic interactions, to a negatively charged phosphate residue. DNA complexes are considered essential materials for numerous applications in photonics and organic molecular electronics [19], including devices based on nonlinear optical effects [20], organic lasers [21], and promising materials in organic light emitting devices (OLEDs) as electron-blocking layers [22]. In our investigations, we have already demonstrated that raw DNA and a DNA complex can stabilise the alignment of mesogenic molecules [12,23,24]. From our perspective, the crucial point was to find an appropriate surfactant and obtain the DNA-based biopolymer alignment layer with the same aligning properties as commercial polyimides. Therefore, the new biopolymer based on DNA and dioctadecyl dimethylammonium (DODA) was studied as a homeotropic alignment layer for liquid crystal devices.

Homeotropic alignment of liquid crystals is fundamentally important for a wide range of photonic technologies, especially when using nematic LCs with negative dielectric anisotropy. In this configuration, the LC director is oriented perpendicular to the substrate, resulting in minimal birefringence in the off-state and enabling high-contrast light modulation. This type of alignment is used in devices such as electrically controlled birefringence (ECB) modulators, optical retarders, and phase-modulating spatial light modulators. Precise control over the director orientation is crucial for achieving targeted switching characteristics and maintaining optical stability [25]. The use of homeotropic alignment in photonic LC devices is widely seen as fundamental to obtaining high-performance and stable optical responses. Homeotropic alignment has long been recognised as a valuable strategy in the design of LC-based photonic devices. One of the industry-standard polyimides that induces homeotropic alignment of liquid crystals and is extensively used in photonic applications is SE-1211 (Nissan Chemical Industries, Ltd., Tokyo, Japan).

For instance, Chychłowski et al. demonstrated that homeotropic alignment using SE-1211 in nematic and chiral nematic phases resulted in significantly more stable, continuous, and energetically favourable molecular configurations than planar alignment, especially in confined geometries like capillaries [26]. This led to clearer optical textures under polarized light and enhanced performance in photonic applications such as tunable lenses and waveguides. Homeotropic alignment in light-induced switching from vertical to planar alignment has also been reported, enabling novel light-controlled modulation schemes [27]. SE-1211 has been employed in wearable photonic devices, such as liquid crystal contact lenses, where its homeotropic properties allow for stable and repeatable electro-optic switching [28]. In smart LC windows, SE-1211 supported thermally activated modulation of transparency, demonstrating consistent performance in dynamic optical applications [29]. Furthermore, it enabled the self-organization of reconfigurable topological defect networks in nematic LCs [30] and was used to investigate LC phases with unconventional optical characteristics [31]. These results confirm the importance of SE-1211 as a reference polyimide in the development of advanced photonic systems.

To explore alternatives to conventional alignment materials with sustainable and functional advantages, a biopolymer-based complex (DNA-DODA) was synthesized using a straightforward procedure. Its structural and surface properties were analysed using multiple experimental techniques, including atomic force microscopy (AFM), Fourier-transform infrared (FTIR) spectroscopy, Raman spectroscopy, and contact angle and surface energy measurements, as well as polarizing optical microscopy. The alignment properties of the DNA-DODA layer were evaluated through electro-optical studies by measuring threshold voltages, anchoring energy, and response times, and its performance was compared to that of a commercial polyimide alignment layer (SE-1211). The presented results demonstrate that the DNA-DODA complex provides effective homeotropic alignment of LC molecules, with anchoring energy and response times comparable to those of traditional polyimides. The material stability and effectiveness in directing light propagation confirmed that the biopolymer is suitable for photonic devices. 

## 2. Materials and Methods

### 2.1. DNA and Surfactant

DNA is considered a linear and unbranched biopolymer whose monomers are deoxyribonucleotides. It consists of biopolymer strands of sugar and phosphate molecules coiled around each other to form a helix linked by hydrogen-bonded base pairs. DNA sodium salt—fabricated from the waste product of the salmon fishing industry—was chosen as a biopolymer source. The claimed molecular weight of DNA sodium salt from Acros Organics (Geel, Belgium) was MW = 50.000–100.000 Daltons (Da) with a purity higher than 96%. The DNA sodium salt layer is too sensitive to water, shows insufficient mechanical strength resistance, and is incompatible with LC devices’ typical fabrication processes. Therefore, we modified the DNA structure using a selected surfactant to make the biopolymer layer more suitable for the production of optoelectronic devices.

**Figure 1:**
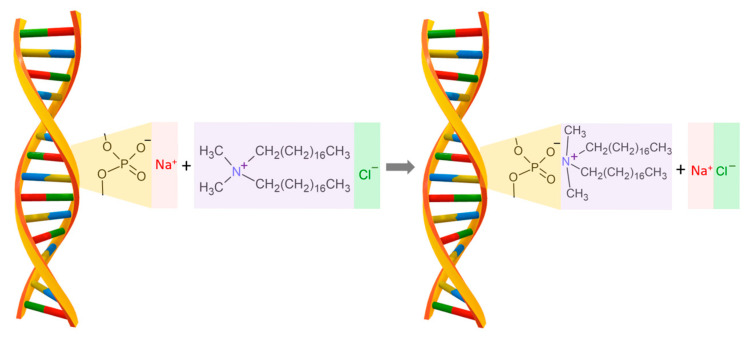
The schematic drawing for the reaction of DNA sodium salt with the DODA surfactant. The final product of the reaction is the biodegradable polymer DNA-DODA, used as an alignment layer in photonic devices.

The cationic surfactant dimethyldioctadecylammonium chloride (CAS Number: 107-64-2, molecular weight of 586.5 g/mol), named DODA, with a purity higher than 97%, purchased from Sigma-Aldrich Co. (St. Louis, MO, USA), was chosen to modify DNA sodium salt. This surfactant was chosen because of its characteristic amphiphilic structure crucial for the synthesis process with DNA sodium salt. DNA forms a complex with a cationic surfactant, such as DODA, using electrostatic interactions between the negatively charged phosphoric acid residue in DNA and the positively charged nitrogen atom in the surfactant. The sodium salt of DNA in an aqueous solution has a negative charge and quickly forms a complex with the cationic surfactant. Both the sodium salt of DNA and the cationic surfactant are soluble in water. In the first step, the surfactant molecule binds via electrostatic interactions with the phosphate residues of the DNA structure, and then the surfactant groups are aligned along the DNA structure due to the strong hydrophobic interactions between the surfactant groups as presented in Figure 1. The resulting DNA–surfactant complex is not soluble in water.

### 2.2. Synthesis of Biopolymer

The synthesis of the biopolymer DNA-DODA was done using the procedure described earlier [25]. DNA sodium salt and the DODA surfactant were dissolved in equal amounts (by weight) in deionised water. Then, the DODA solution was added to the DNA sodium salt solution drop-by-drop on a magnetic stirrer. The precipitated DNA-DODA complex was filtered from solutions, dried at room temperature, and dissolved in butanol (3%).

### 2.3. Liquid Crystal

The created biodegradable DNA-DODA polymer was used to align liquid crystal molecules. The liquid crystals (LCs) are self-organised compounds that were used in this research as the optically active medium [25]. LC, as a type of soft matter, combines crystalline-like solid ordering with fluid-like behaviour and is attractive for many display and photonic devices’ technology [32].

**Figure 2:**
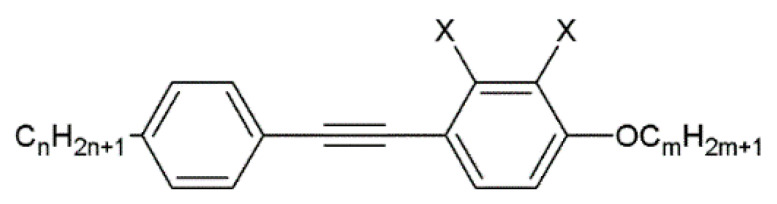
The chemical structure of organic compounds used in the 1832 nematic mixture. X = -H or -F, n = 3–6, and m = 1–4.

A nematic liquid crystal is a specific compound with an achiral phase representing a purely orientational order of elongated molecules desired for homeotropic or planar aligning. To verify a proper alignment layer of the biopolymer complex, an 1832 nematic mixture (synthesized at the Military University of Technology), with a negative dielectric anisotropy of Δε = −1.1 at frequency 1 kHz, was used. This mixture exhibits good properties, beneficial for photonic with optical anisotropy Δn = 0.23 (at 589 nm, 20 °C) and short switching times in the order of milliseconds. The mixture consists of 1-alkoxy-4-[(4-alkyl phenyl)ethynyl] benzene and 2,3-difluoro-1-alkoxy-4-[(4-alkyl phenyl)ethynyl] benzene with different alkoxy and alkyl chain lengths. Those compounds are represented by the general formulas presented in Figure 2.

### 2.4. Preparation of the Alignment Layer

A typical testing cell for alignment layer properties comprises two sandwiched glass plates covered by an indium tin oxide (ITO) conductive layer and a thin polymer aligning layer. The properties of the polymer layer surface dictate the LC molecules’ preferred orientation at the interface by some specific agent: dipolar interactions, chemical bonding, Van der Waals interactions, steric factors, and surface topographies [33]. To obtain a durable biopolymer alignment layer, the same fabrication procedure was used for cells with the commercial polyimide alignment layer as for DNA-DODA. The DNA-DODA complex was spin-coated onto a glass/ITO substrate at a speed of 3500 rpm for 30 s. The coating was then annealed to evaporate the solvent at a temperature of 135 °C for 90 min. In the next step, two substrates were glued together. To control the cell thickness, 5 µm glass spacers were used. Nematic liquid crystal at the isotropic phase was injected into the cell by capillary forces.

### 2.5. Atomic Force Microscopy

The atomic force microscopy (AFM) technique was implemented to visualize the topography of three types of materials: DNA sodium salt, DODA, and DNA-DODA. The research was done using Bruker’s Dimension Icon AFM system. Before the investigation, a silicon AC200TS scanning probe (Olympus, Tokyo, Japan) was installed, and its drive frequency (ca. 139 kHz) was calibrated using the Auto-Tune built-in function. The probe operated in Tapping Mode at room temperature (21 °C) and a relative air humidity of approximately 38%. Topographical images were recorded over three randomly selected areas for each sample. These images were used for a qualitative description of the tested surfaces, and subsequently, based on these images, a quantitative analysis of the average roughness parameter Ra was performed using the Gwyddion software (ver. 2.56).

### 2.6. Spectroscopy

Attenuated Total Reflectance Fourier Transform Infrared Spectroscopy (ATR FT-IR, ALPHA FT-IR Spectrometer, Bruker Optics, Ettlingen, Germany) was used to characterise DNA sodium salt, DODA, and DNA-DODA. The spectra were recorded using a diamond crystal within a wavenumber range of 400–4000 cm^−1^ with a resolution of 2 cm^−1^ from 24 scans. The spectra are average spectra from 5 random points on the surface of each sample. The spectra were imported via the OPUS software 7.5 (Bruker, Ettlingen, Germany).

The Raman spectra of DNA sodium salt, DODA, and DNA-DODA were acquired using a Renishaw inVia Reflex Raman microscope (Renishaw Plc, Gloucestershire, UK) equipped with an EMCCD detector (Andor Technology Ltd., Belfast, UK). The measurements were carried out using laser radiation with a wavelength of 532 nm, laser power of ca. 10 mW, and Leica objective lens 100×. The other measurement parameters were an acquisition time of 1 s and 20 accumulations at each point. The Raman spectra were acquired from at least 100 points. The spectrometer was calibrated using an internal silicon wafer. All collected spectra were processed in the WiRE 5.5 software and then averaged using the CasaXPS software version 2.3.17 (Casa Software Ltd., Teignmouth, UK).

### 2.7. Contact Angle Measurements

Contact angle (CA) measurements were made using a Mobile Surface Analyzer (MSA) from Krüss company (Hamburg, Germany). The static sessile drop method was used for two liquids: water (polar liquid) and diiodomethane CH_2_I_2_ (non-polar liquid). The volume of each drop was 2 µL. In addition, the surface free energy (SFE) was determined using the Owens, Wendt, Rabel, and Kaelble (OWRK) model [34]. The OWRK model divides SFE into two components: dispersive and polar. Wettability and surface free energy parameters were collected from at least 12 points.

### 2.8. Electro-Optics

Electro-optical measurements were carried out to confirm that the fabricated biopolymer film can be used with success in photonic devices as an alignment layer. In the experiment, the LC cells were placed between the crossed analyser and polarizer of the polarizing optical microscope (POM). To investigate the change in the intensity of light passing through the cell, white light with an optical filter of 589 nm as the incident light source was used. An AC electric field (0–20 V, 1 kHz, sinusoidal wave) was applied to the cells by a function generator (Hewlett-Packard 33120A) and monitored by an oscilloscope (Hewlett-Packard 346013). The light intensity was recorded using a linear optical detector. The capacitance of the cells was measured using the impedance analyser (Hewlett-Packard 4284A) at a constant frequency of 1.5 kHz. All electro-optical measurements were conducted at room temperature (23 °C).

## 3. Results

**Figure 3:**
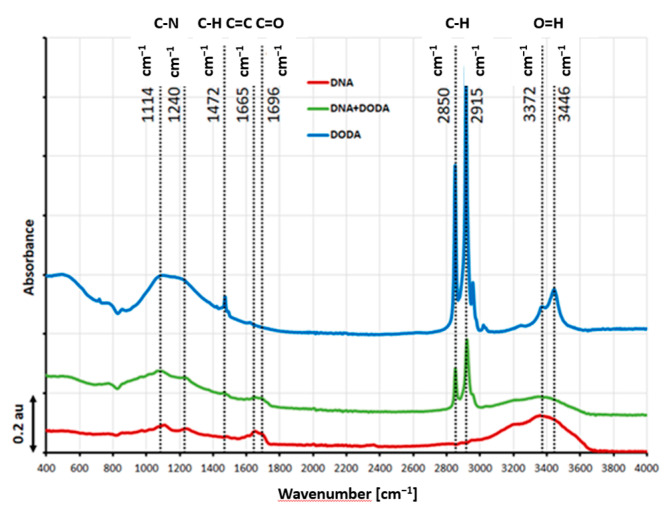
ATR FT-IR baseline-subtracted spectra of DNA (red line), DODA (blue line), and DNA-DODA (green line). As a baseline, the glass substrate was measured.

The FTIR spectra of the DNA, DODA, and DNA-DODA layers are depicted in Figure 3, showing several distinct vibration bands. The bands visible in the pure DNA spectrum have been reported in the literature as characteristic features [35]. Notably, a broad absorption band spanning 3000–3400 cm^−1^ can be attributed to various molecular vibrations, including N-H stretching modes, C=N vibrations, and O-H symmetric and antisymmetric stretching modes. Compared to the pure DNA spectrum, the following absorption bands appear in the DNA-DODA spectrum: 2915 and 2850 cm^−1^. These bands correspond to symmetric and asymmetric stretching C-H vibrations of the -CH2 and -CH3 groups, which is in complete agreement with the band present in the spectrum of the pure surfactant (DODA). It should also be noted that the visible bands at 3371 and 3446 cm^−1^ (O-H stretching modes) in the DODA spectrum are evidence of the presence of water remaining after the layer formation process.

**Figure 4:**
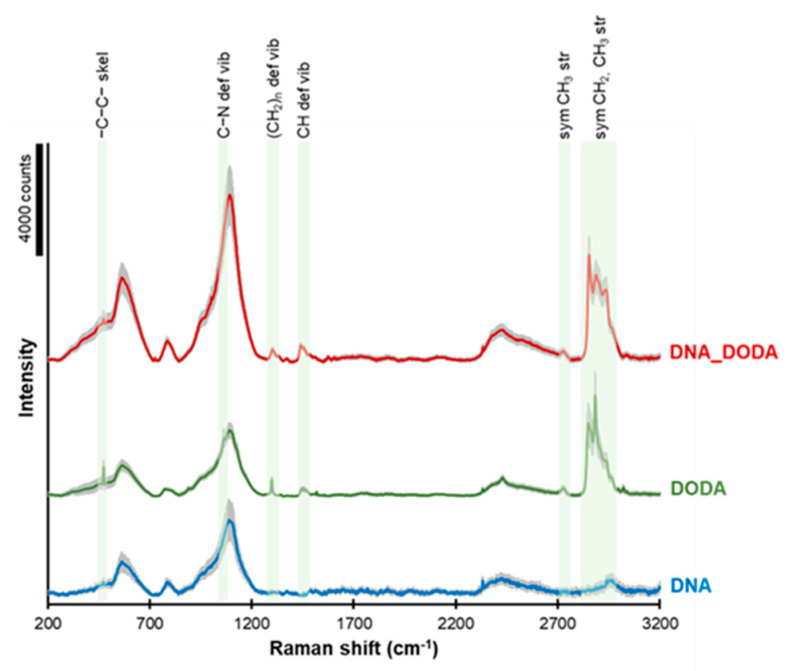
Raman spectra of DNA (blue line), DODA (green line), and the DNA-DODA complex (red line). A grey area in each spectrum shows the standard deviation of the signal.

The Raman spectra of DNA, DODA, and the DNA-DODA complex are presented in Figure 4. The green areas indicate characteristic bands from the DODA surfactant. The peaks observed at ca. 2840–2970, 2730, 1450, 1300, 1090, and 475 cm^−1^ correspond to symmetric CH2 and CH_3_ groups’ stretching, symmetric CH_3_ stretching, CH deformation vibration, (CH2)_n_ deformation vibration, C-N deformation vibration, and -C-C- skeletal vibration, respectively. These peaks completely agree with the bands observed in the FT-IR spectrum, which is a complementary method for studying molecular oscillations. The presence of these peaks in both the DODA and the DNA-DODA spectra confirms that the DODA surfactant has indeed bound to the DNA in the DNA-DODA sample.

**Figure 5:**
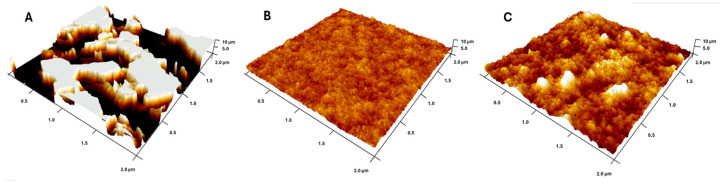
AFM topographical images of tested samples: (**A**) DODA, (**B**) DNA sodium salt, (**C**) DNA-DODA. The scanning area was 2 × 2 µm.

To gain deeper insight into the wetting behaviour of the prepared surfaces, we analysed the correlation between surface roughness and water contact angle, both of which are influenced by surface structure and chemistry. The topography visualization of the biopolymer alignment layers using AFM (Figure 5) revealed distinct morphological features among the tested materials. The pristine DODA surface was characterized by terrace-like structures formed by stacked, flat layers (Figure 5A), whereas the DNA and DNA-DODA films (Figure 5B,C) exhibited smooth and homogeneous topographies without specific features.

Quantitative roughness analysis showed that the DODA surface had the highest average roughness (Ra = 3.87 ± 0.73 nm), while DNA and DNA-DODA exhibited lower values of 0.21 ± 0.01 nm and 0.42 ± 0.01 nm, respectively. Notably, the DNA-DODA layer was approximately twice as rough as pristine DNA, indicating a significant impact of surfactant integration on nanoscale morphology. These structural differences strongly influenced the wettability of the layers.

**Figure 6:**
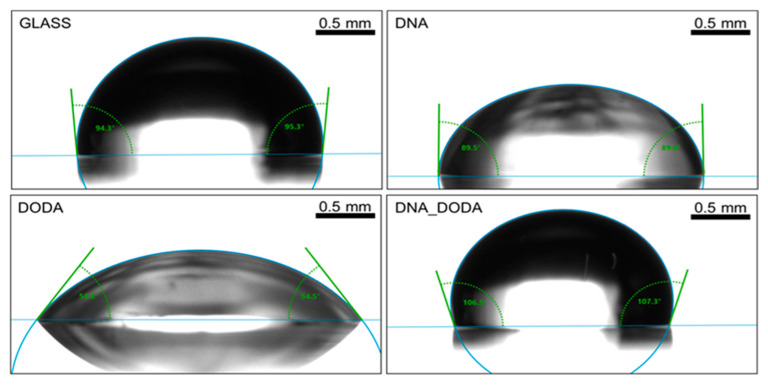
Contact angle measurements for water on the glass, DNA, DODA, and DNA-DODA complex surfaces.

**Figure 7:**
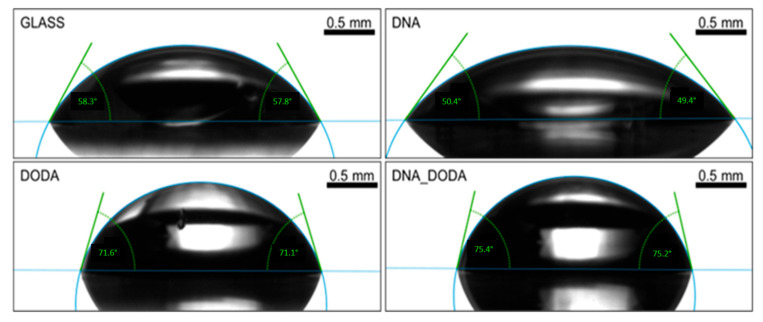
Contact angle measurements for diiodomethane on glass, DNA, DODA, and DNA-DODA complex surfaces.

As presented in Figure 6, the DNA-DODA film showed the highest contact angle for water (106.9°), while pristine DNA exhibited a moderate angle of 89.8°, and DODA showed the lowest value (54.3°). Figure 7 shows the results for diiodomethane, which followed a similar trend, supporting the interpretation based on surface free energy components.

These findings are consistent with the Wenzel model, which predicts that on hydrophobic surfaces, increased surface roughness amplifies water repellency by enhancing the effective contact area and promoting air entrapment [36]. However, this effect does not operate in isolation: despite its high roughness, DODA exhibited strong hydrophilic behaviour. This apparent contradiction highlights the overriding role of surface chemistry, specifically the presence of polar functional groups such as quaternary ammonium headgroups in DODA, which decrease contact angles irrespective of topography.

Such dual dependence on roughness and chemistry has also been observed in inorganic systems. For example, Kim et al. [37] demonstrated that in silicon carbide wafers, moderate roughness could reduce contact angles due to increased polar interactions—a phenomenon comparable to what we observe in DODA. Moreover, Razavifar et al. [38] conducted a systematic analysis of surface roughness and wettability, showing that for surfaces with low polar energy, increasing roughness enhanced hydrophobicity—supporting our observations for DNA-DODA. Conversely, for polar surfaces, the effect was reduced or even reversed, as in the case of DODA.

Surface energy data (Table 1) further corroborate this interpretation. DNA-DODA exhibits a very low polar component (0.51 mN/m), making it highly hydrophobic, while DNA retains a substantial polar character (34.28 mN/m). DODA, although structurally rough, maintains relatively high surface energy due to its strong polar interactions. These results collectively confirm that wettability in biopolymer-based alignment layers is governed by a complex interplay between topography and surface chemistry, fully consistent with both classical models and recent experimental studies.

The usefulness of the biopolymer DNA-DODA as an alignment layer was tested in the electronically controlled birefringence effect (ECB) [39].

**Figure 8:**
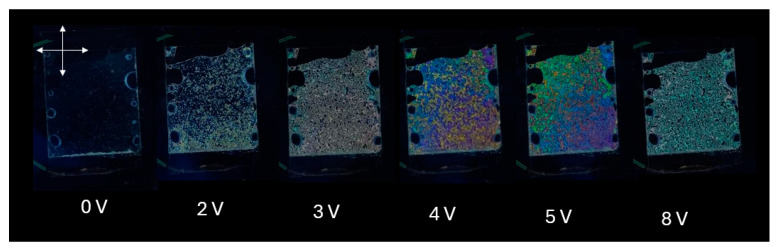
Optical macroscope images of a homeotropically aligned nematic liquid crystal cell with a DNA-DODA layer placed between crossed polarizers at 0–8 V.

Figure 8 presents a series of macroscopic polarized optical microscopy images of a liquid crystal (LC) cell with homeotropic alignment, fabricated using a DNA-DODA alignment layer, subjected to various applied voltages (0 V, 2 V, 3 V, 4 V, 5 V, and 8 V). The cell was placed between crossed polarizers and illuminated with white LED light.

In the ECB effect, the liquid crystal director n, which describes the average molecular orientation, is aligned homeotropically without an external electric field, perpendicular to the glass substrates. This configuration yields a dark state under crossed polarizers because the effective refractive index Δn_eff_ of the LC is the same for all incident polarization components, resulting in no birefringence and thus complete extinction of light.

The phase retardation Γ introduced by the birefringent LC layer is given as follows:

(1) $$\Gamma =\frac{2\pi d{\Delta n}_{\mathrm{eff}}}{\lambda }$$

where d is the thickness of the LC layer, and λ is the wavelength of the incident light.

The effective birefringence in the ECB mode can be approximated as follows:

(2) $${\Delta n}_{eff}=n_{e}n_{o}\left(\frac{1}{\sqrt{{\left(n_{o}\sin\;\theta \right)}^{2}{+\left(n_{e}\cos\;\theta \right)}^{2}}}-\frac{1}{n_{o}}\right)$$

where n_o_ and n_e_ are the ordinary and extraordinary refractive indices of the liquid crystal, and θ is the angle between the LC director and the substrate normal. The electric field E = V/d induces increasing molecular tilt, which increases the effective birefringence thereby enhancing the optical response of the LC cell under crossed polarizers

As the voltage increases, starting from 2 V, slight optical retardation begins to appear, visible as faint colour changes, indicating the onset of molecular tilt. At 3 V, a more pronounced texture emerges due to spatial variations in the molecular orientation. From 4 V to 5 V, the birefringence becomes increasingly significant, giving rise to colourful interference patterns caused by the phase retardation of different wavelengths of white light. At 8 V, the texture becomes more saturated and uniform, suggesting a near-saturation of the molecular tilt, with most molecules reoriented closer to the substrate plane.

These optical changes occur because the application of an electric field to a nematic liquid crystal with negative dielectric anisotropy (Δε < 0) induces reorientation of the director from the homeotropic (perpendicular) to a more planar (parallel) configuration. This reorientation modifies the optical axis and introduces birefringence. As a result, the LC cell transitions from a dark to a bright state under crossed polarizers. The voltage-dependent change in birefringence Δn leads to phase retardation, which causes wavelength-selective interference effects and produces voltage-dependent colour variations.

**Figure 9:**
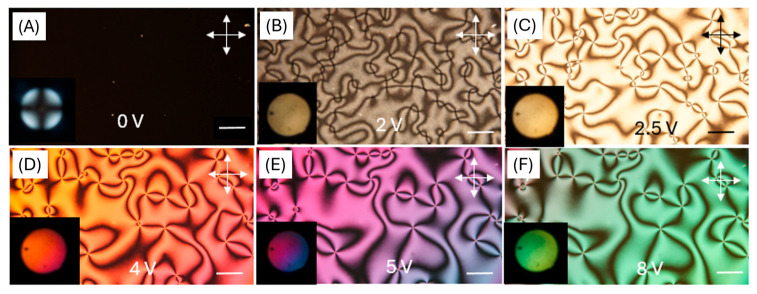
Orthoscopic and conoscopic polarizing microscope images of the LC cell with a DNA-DODA alignment layer, placed between crossed polarizers. (**A**) Optical textures at 0 V; homeotropic alignment. (**B**–**F**) Optical textures at 2–8 V; gradual reorientation of LC molecules under applied voltage. Photos in the lower left corners show the conoscopic images.

The quality of liquid crystal ordering was confirmed using the orthoscopic and conoscopic modes of POM. Conoscopic observations confirm perfect homeotropic ordering by observing the classical “Maltese cross” due to symmetrical interference (Figure 9A) [40]. The optical quality of alignment was at least comparable to, and in some cases more uniform than, that achieved with the commercial polyimide SE-1211, as reported in our previous work [12]. After applying voltage, non-uniform textures can be observed, as in Figure 9B–F. The presented textures are typical for the nematic phase and show that liquid crystal molecules arrange themselves in a random direction under the influence of an electric field.

**Figure 10:**
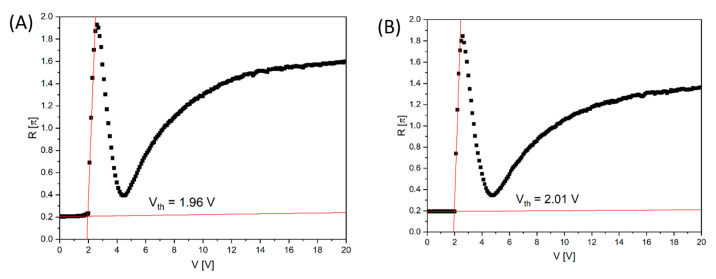
The voltage-dependent phase retardation R of homeotropically aligned LC cells prepared using two different surfactants: (**A**) commercial polyimide SE-1211 and (**B**) biopolymer DNA-DODA. The calculated threshold voltage Vth from the linear extrapolation is 1.96 V and 2.01 V for SE-1211 and DNA-DODA cells, respectively.

In the next step, we checked whether the cells prepared using the biopolymer and conventional polyimide would switch equally under the influence of the applied voltage and whether they would be equally susceptible to the action of an electric field. The phenomenon of director switching is called a Frederick transition and it takes place when the value of the external electric voltage exceeds a minimum so-called threshold voltage, Vth [41]. From the light transmittance measurement of the liquid crystal cell under the applied voltage V, the phase retardation R can be obtained from the following formula [42]:

(3) $$R=2\arcsin\left(\sqrt{\frac{I}{I_{0}}}\right)$$

where I_0_ is the incident light intensity and I is the intensity of light coming out of the cell between crossed polarizers. In the experiment, the optical axis of the homeotropically aligned cell with negative dielectric anisotropy liquid crystal is oriented at 45° with respect to the polarizer. Figure 9 shows the voltage-dependent retardation change at λ = 589 nm and T = 23 °C. The behaviour of the electro-optical response is similar for the cells made with the commercial polyimide SE-1211 (Figure 10A) and the biopolymer DNA-DODA (Figure 10B), and as ordering alignment layers. At voltages below the threshold voltage value Vth, the molecular orientation of LC becomes undistributed, and no phase retardation is observed. The change of molecular orientation starts at the Vth. The threshold voltage value was determined from the extrapolation method [43]. From Figure 10A,B, Vth is found to be 1.96 V and 2.01 V for SE-1211 and DNA-DODA, respectively. The results confirm that the biopolymer DNA-DODA does not cause a significant increase of Vth. The small difference between the values is at the level of a measurement error. We observed a slight deviation from the perfect homentropic alignment in the case of the commercial alignment layer because the transition threshold is not very sharp. In the case of the DNA-DODA material, the pretilt angle is close to 90°. The slight voltage differences in the electro-optical characteristics for both alignment layers are probably due to differences in the anchoring energies of the LC molecules on both orientation layers.

To further analyse the electro-optical properties of the studied materials, we calculated the polar anchoring energy W of homeotropic cells using the biopolymer DNA-DODA and polyimide SE-1211. The anchoring energy is a crucial parameter for liquid crystal-based photonics devices. It influences not only the orientation of molecules on the surface but, above all, the threshold voltage and the on and off response times. Based on the voltage V-dependent capacity C and retardation R of light passing through the liquid crystal cell, we precisely determined W.

**Figure 11:**
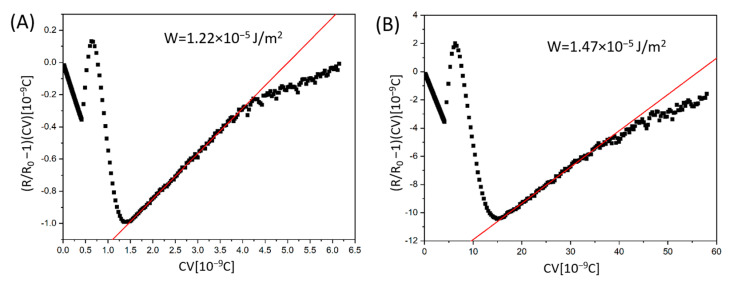
The determination of anchoring energy W from the slope fitting method, according to [36]. The plots were obtained in the homeotropically aligned cells prepared using commercial polyimide (**A**) SE-1211 and (**B**) biopolymer DNA-DODA.

The anchoring energy can be determined from the slope of a plot, (R/R_0_ -1)CV, as a function of CV, where R_0_ is maximum phase retardation. Figure 11A,B presents the linear slope fitting method obtained for the DNA-DODA and SE-1211 cells, respectively. The results indicate a good linear fit. The plot is the linear function in a specific voltage regime, from around 7 to 13 V. Calculation of the slope coefficient of straight lines allowed the determination of the W parameter, taking into account the bend elastic constant K_33_ = 8.6 pN of 1832 and thickness of the measuring cell, d [44]. For the 5 μm-thick cell prepared using the commercial rubbed polyimide SE-1211, W is calculated to be 1.22∙× 10^−5^ J/m^2^. The obtained anchoring strength for the biopolymer DNA-DODA is W = 1.47 × 10^−5^ J/m^2^, in a 4.6 μm-thick cell. Comparing these two values, it can be seen that the anchoring energy for both investigated materials is very similar. From the point of view of the molecular interactions between the studied surfactants and the polar molecules of the liquid crystal, there is no significant difference between DNA-DODA and SE-1211. To further situate the performance of DNA-DODA within the broader alignment techniques, we compared the anchoring energy values with those typically observed in photoalignment systems. Photoalignment layers, while allowing non-contact and high-resolution patterning, generally provide lower anchoring energies in the range of 10^−6^ to 10^−7^ J/m^2^ [45,46]. In contrast, the DNA-DODA layer demonstrated significantly higher anchoring energy (1.47 × 10^−5^ J/m^2^), confirming stronger interfacial coupling and improved electro-optic stability.

**Figure 12:**
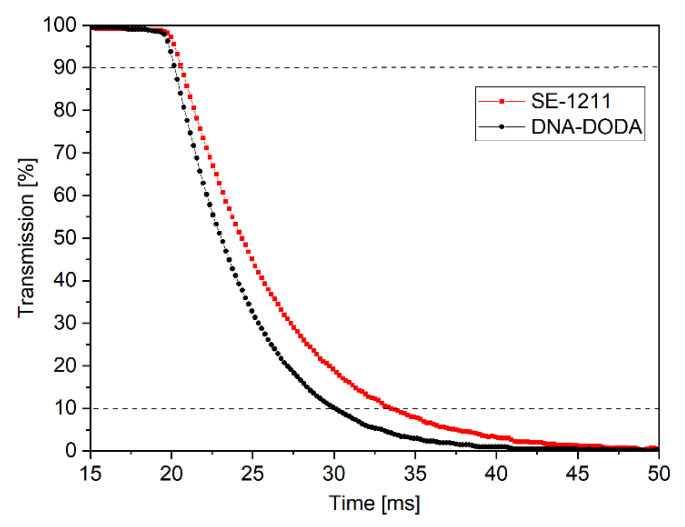
Switching-off times of liquid crystal cells with DNA-DODA and SE-1211 alignment layers.

The anchoring energy is a critical factor in determining the time response of liquid crystals. Therefore, LC switching times were measured in the next step of the study. Figure 12 shows the electro-optical characteristics of LC cells with a DNA-DODA and a commercial SE-1211 alignment layer. These characteristics determined the turn-off times (i.e., the time during which the LC cell transmission changes from 90% to 10% after the electric field pulse disappears). The turn-off times were measured at the first transmission peak (i.e., δ = π), V = 2.5 V (1 kHz, square signal). The switching on time is the same (ton = 1 ms) for all investigated cells because the switching mechanism in the ECB effect depends mainly on the applied electric field [32]. However, the turn-off mechanism is related to the liquid crystal medium and alignment layer properties. Based on the time response measurements, it can be concluded that the turn-off times were t_off_ = 10 ms for the cell with DNA-DODA layers and t_off_ = 13 ms for the cell with SE-1211 layers. The experiments show that the liquid crystal cell with the DNA-DODA alignment layer possesses a comparable response time to the commercially available cell. Therefore, based on the presented studies, we proved that the DNA-DODA complex can be successfully used in liquid crystal transducers.

As demonstrated by our electro-optical measurements, the DNA-DODA alignment layer offers performance comparable to the commercial SE-1211 material, including a low threshold voltage, rapid switching, and strong anchoring energy. These results confirm that the biopolymer DNA-DODA is not only functionally effective but also environmentally advantageous.

Given these attributes, the DNA-DODA complex holds promise for various next-generation photonic applications, including biodegradable optical components for disposable or eco-sensitive systems, biocompatible wearable devices such as contact lenses and skin-mounted displays, as well as transient and flexible photonics designed to minimize environmental persistence after use. Additionally, the increased hydrophobicity and reduced surface energy of the DNA-DODA surface support device reliability under humid or biologically active conditions, enabling its use in outdoor, in vivo, or portable systems.

This compatibility between performance and sustainability suggests that DNA-DODA may serve as a viable platform for replacing synthetic alignment layers in mainstream photonic manufacturing.

While the main scope of this study focused on the performance of the DNA-DODA alignment layer in combination with nematic liquid crystals (LCs), it is important to acknowledge that the overall electro-optic performance of LC-based devices also strongly depends on the intrinsic properties of the LC material itself. Ferroelectric liquid crystals (FLCs), which exhibit spontaneous polarization and bistable switching, can achieve response times in the microsecond range—significantly faster than the millisecond-scale switching of nematic LCs. These properties make FLCs highly attractive for high-speed applications such as optical shutters, reflective displays, and spatial light modulators, where rapid response, high contrast, and high resolution are essential [47,48].

However, the alignment requirements for FLCs differ fundamentally from those of nematic LCs. Smectic C* phases, commonly used in FLC devices, require specific alignment geometries such as bookshelf or chevron structures, typically induced by tilted anchoring. In contrast, the DNA-DODA layer presented in this study has been specifically optimized for inducing robust homeotropic alignment in nematic LCs, which is ideal for applications such as ECB modulators and tunable optical retarders.

However, recent studies have demonstrated that surface-engineered or photoresponsive layers can be modified to enable FLC alignment. For instance, photoalignment methods using azobenzene dyes or functionalized polymers have successfully induced stable bookshelf geometries in FLCs, with controllable pretilt angles and enhanced switching behaviour [49]. These strategies could potentially be adapted to bio-based systems such as DNA-DODA through molecular engineering or hybrid functionalization. Further research in this direction may enable the expansion of sustainable alignment layers into more complex LC technologies beyond the nematic phase.

## 4. Conclusions

The proposed biopolymer complex from fish waste was simply synthesized and showed availability as an alignment surface promoting alignment of LC molecules. The DNA-DODA complex as an alignment surface promotes a vertical alignment of LC molecules, which are perpendicular to the surface and maintain the stable orientation of neighbouring molecules. Our research has proven that a DNA-based complex could be a good alternative for LC materials as a non-conventional alignment layer for developing photonic devices. In addition to its electro-optical performance, the DNA-DODA alignment layer offers clear ecological benefits compared to conventional polyimides. Unlike synthetic PI materials, which typically require harsh chemical synthesis involving toxic solvents and high-temperature processing, DNA-DODA is derived from renewable fish-waste biomass and processed under relatively mild conditions. Its biodegradable nature reduces the environmental impact at the end of the device lifecycle, making it a more sustainable alternative. These features suggest that DNA-DODA may serve as a promising alignment material for future eco-conscious photonic and display technologies, particularly in applications requiring biocompatibility or green manufacturing strategies

## Figures and Tables

**Table 1:** Experimentally determined parameters of layer wettability and surface free energy.

Sample	SFE [mN/m]	Disperse [mN/m]	Polar [mN/m]	CA H_2_O [°]	CA CH_2_I_2_ [°]
GLASS	31.00 ± 2.03	29.58 ± 2.12	1.42 ± 0.61	95.02 ± 4.90	58.25 ± 3.69
DNA	36.34 ± 1.78	2.06 ± 0.63	34.28 ± 1.21	89.79 ± 7.16	49.98 ± 2.15
DODA	47.08 ± 1.78	22.14 ± 0.31	24.94 ± 1.92	54.33 ± 2.53	71.32 ± 0.56
DNA-DODA	20.46 ± 0.26	19.96 ± 0.23	0.51 ± 0.23	106.90 ± 1.75	75.31 ± 0.43

## Data Availability

Data supporting the reported results are available from the corresponding author upon reasonable request.

## Abbreviations

The following abbreviations are used in this manuscript:

AFM	Atomic force microscopy
ATR FT-IR	Attenuated Total Reflectance Fourier Transform Infrared Spectroscopy
CA	Contact angle
DODA	Dimethyldioctadecylammonium chloride
DNA	Deoxyribonucleic acid
ECB	Electrically controlled birefringence
FTIR	Fourier Transform Infrared Spectroscopy
ITO	Indium tin oxide
LC	Liquid crystal
OLED	Organic light emitting device
POM	Polarizing optical microscopy
Ra	Average surface roughness
SFE	Surface free energy
Vth	Threshold voltage
